# Low Genetic Variability of the Tundra Vole in Lithuania

**DOI:** 10.3390/ani14020270

**Published:** 2024-01-15

**Authors:** Petras Prakas, Dalius Butkauskas, Laima Balčiauskienė, Linas Balčiauskas

**Affiliations:** Nature Research Centre, Akademijos Str. 2, LT-08412 Vilnius, Lithuania; dalius.butkauskas@gamtc.lt (D.B.); laima.balciauskiene@gamtc.lt (L.B.); linas.balciauskas@gamtc.lt (L.B.)

**Keywords:** *Alexandromys oeconomus*, *cytb*, control region, genetic variability, natural and anthropogenic barriers, phylogeography

## Abstract

**Simple Summary:**

The tundra vole’s (*Alexandromys oeconomus*) distribution in Lithuania has been documented for 70 years, yet its genetic diversity remains unexplored. We analyzed vole samples from northern and western sites in Lithuania, using mtDNA sequence analysis. Despite landscape barriers, our phylogenetic analyses placed Lithuanian voles in the Central European phylogroup, suggesting an origin from northeastern Poland. Genetic diversity in Lithuanian *A. oeconomus* at the mtDNA loci was low compared to other European samples, revealing distinctions from Poland and Northern Europe. Genetic divergence among western and northern Lithuanian samples, coupled with low variability, provides novel insights into species phylogeography and the influence of barriers on colonization dynamics.

**Abstract:**

The distribution and spread of the tundra vole (*Alexandromys oeconomus*) in Lithuania have been documented over the last 70 years, but the genetic diversity of the species has not been studied. In this study, we examined *A. oeconomus* trapped in three sites in northern and western Lithuania using mtDNA sequence analysis of the *cytb* and control region. The western and northern sites are separated by anthropogenic landscape barriers. The western site is subject to regular spring flooding. Phylogenetic analyses of the studied individuals placed them in the Central European phylogroup, suggesting that Lithuanian *A. oeconomus* originated from northeastern Poland. In Lithuania, the genetic diversity of *A. oeconomus* at both mtDNA loci was relatively low (*Hd* < 0.6, π < 0.002) compared to that found in other European samples (*Hd* = 0.833–0.958; π = 0.00402–0.01552). Individuals analyzed in Lithuania were genetically different from samples collected in Poland and Northern Europe (Φ_ST_ > 0.15, *p* < 0.05). The genetic divergence between the western and northern samples of *A. oeconomus* in Lithuania, together with the low genetic variability among the voles studied, provides new insights into the phylogeography of the species and the influence of barriers on the colonization of the country.

## 1. Introduction

Recent studies have shown that genetic diversity is currently declining in many mammalian populations, with lower heterozygosity in populations under demographic threat [1]. Different perspectives on the genetic diversity of European vole species are related to their phylogeographic history—recolonization after the last glacial period [2]. In the common vole (*Microtus arvalis* (Pallas, 1778)), multiple glacial refugia have led to different genetic lineages [3], a similar characteristic pattern to the bank vole (*Clethrionomys glareolus* (Schreber, 1780)), and widespread forest habitation [4]. In the field vole (*Microtus agrestis* (Linnaeus, 1761)), three main genetic groups were found [5], with two of these being present in Lithuania [6].

Voles of the genus *Microtus* represent one of the most speciose mammalian genera in the Holarctic [7]. Over the last two million years, the genus has rapidly evolved into a group of 65 species, spread over a wide range of latitudes [8,9]. The most widespread species is the tundra vole (*Alexandromys oeconomus* (Pallas, 1776)). However, not all investigations recognize the validity of the subgenus *Alexandromys* [7], so this species is also recognized as *Microtus oeconomus* (Pallas, 1776) [9,10,11]. Here, we use *A. oeconomus*, following the nomenclature proposed by the American Society of Mammalogists and IUCN [12,13].

*Alexandromys oeconomus* survived glaciation in the northern refugia in Norway [14]; therefore, the species recolonization pattern could be somewhat different from other *Microtus* voles [3,5]. The species is shown to be absent in southern parts of Finland and Sweden, and in the St. Petersburg region, nearly absent in Estonia and, has been mistakenly reported to be absent in Latvia [12,15].

The presence of two isolated *A. oeconomus* subspecies, namely *A. o. mehelyi* (Èhik, 1928) from the Pannonial lowland, and *A*. *o*. *arenicola* (de Sélys-Longchamps, 1841) from the Netherlands [12,16,17,18], sparked scientific and conservationist interest on the genetics of this species.

Following phylogeographical study of the species based on the 1140 base pairs (bp) of the mitochondrial cytochrome b (*cytb*) gene, C. Brunhoff et al. [19] identified four main mitochondrial DNA (mtDNA) phylogenetic lineages: the Beringian, the Central Asian, the North European, and the Central European. Lithuania, in this study, was represented by only one individual, related to the Central European mtDNA phylogroup. The authors additionally implied that during the last glacial period, *A. oeconomus* also survived in areas north of the classical refugial areas in southern Europe. This study was a frame for our investigation to obtain more information on the genetic diversity of this species in Lithuania. No such investigations have been carried out in the country after 2003. Initially, low genetic differentiation was found not only in northwestern Europe but also in other populations. As an explanation for this, multiple events of population decrease during and after glaciation, based on a severe reduction in the suitable habitat, were assumed [20]. Later, lower genetic diversity was also confirmed for *A. o. mehelyi* [17], differing from the most widely distributed *A. o. stimmingi* (Nehring, 1899).

Another reason for the low genetic diversity of *A. oeconomus* is the isolation of current populations, especially in the Netherlands [16] and Hungary [18]. Isolation due to distance has not been found to affect the genetic differentiation of isolated populations in the Danube Delta [21], so these authors attribute the low genetic diversity to the isolated location of the whole area in an agricultural landscape. Long-term habitat changes in the Kis Balaton area in Hungary have resulted in the isolation and low genetic variability of *A. oeconomus*, which differs from other Hungarian, Austrian, and Slovak populations [18]. However, data on the genetic structure of *A. oeconomus* in Poland were different [10]. The Polish authors found that isolation due to distance is a major factor in genetic differentiation. While natural barriers can be overcome due to the migratory capacity of the species, anthropogenic barriers can have a much stronger effect [10].

The genetic diversity of any population under study can be related to the history of the population. In the Bialowieza Primeval Forest, *A. oeconomus* has been continuously present for a long period of time in the undisturbed marshes, so a high genetic diversity should be expected. Indeed, eight mtDNA *cytb* haplotypes, with four of these being new to the species, were identified [22]. According to the haplotype network analysis, two of these haplotypes were very important: PLB5 confirmed the link between the indigenous Bialowieza population and the Northern European populations, while PLB8 was linked to a number of Central European haplotypes. Genetically, the population of *A. oeconomus* in the Bialowieza Forest is stable, although the number of voles has changed fourfold in a short period of time [23]. The genetic stability of many populations that maintain abundance over long periods has also been confirmed in Northeast Asia and Alaska [24].

In Lithuania, *A. oeconomus* (*M. oeconomus* in all publications mentioned below) was first recorded in 1949–1950 in the Nemunas delta, in the western part of the country, and less than 100 km along the Nemunas River to the east [25]. In the 1950s, the species appeared in two strict nature reserves located in the southern and southwestern parts of Lithuania, which are relatively close to eastern and northeastern Poland [26]. Therefore, migration from the Polish population to Lithuania was presumed. This part of Poland has strong populations of the species [27] as a result of postglacial dispersal and configuration of hydrogenic habitats [28]. In the 1990s, the largest number of registrations was in southern and southwestern Lithuania. Over 50 years, a further spread of the species towards the northeast has been documented [29]. By contrast, there is only one record of this species being present in Latvia so far [15]; therefore, the Lithuanian population is at the edge of the species’ continuous range.

The main habitats of the species in Lithuania include flooded meadows and wetlands, often tending toward reedbeds. The numbers of trapped animals have also been high in wet forests [30]. In smaller numbers, *A. oeconomus* has been trapped in various other habitats, such as farmsteads, fruit gardens, and ecotones of agricultural fields. In northern Lithuania, *A. oeconomus* was exceptionally trapped in natural meadows [31]. During natural or human-induced succession when meadows were overgrown with forest, *A. oeconomus* disappeared [32].

While knowledge of *A. oeconomus* distribution, reproduction, and ecology in Lithuania is relatively well known [29,30,33], no investigations have been conducted into its genetics so far. Therefore, the genetic diversity of *A. oeconomus* in Lithuania, a species on the edge of a continuous distribution range, is of interest. The aim of the study was to assess the genetic variability and the population structure of *A. oeconomus* using mtDNA *cytb* and control region sequence analysis in Lithuania and compare these with the populations in other European countries. Taking into account the history of the species, its distribution in the country, and its affinity to wet habitats, we expected that the genetic diversity of *A. oeconomus* in Lithuania would be low. We also aimed to confirm whether the Lithuanian population of *A*. *oeconomus* originated from northeastern Poland.

## 2. Materials and Methods

### 2.1. Small Mammal Sampling

**Figure 1:**
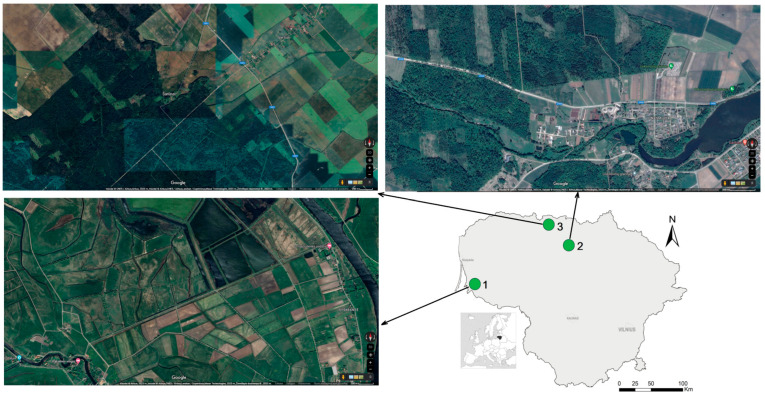
Sampling sites and their habitat structure in Lithuania.

Samples of *A. oeconomus* were collected from three sites in Lithuania (Figure 1). Small mammals in Site 1, in the western part of the country near the Rusnė settlement (55.324° N, 21.339° E), were trapped in October 2011 and September 2012, yielding 24 individuals. Sites 2 and 3 were located in the northern part of the country. Site 2, near the Linksmučiai settlement in the Pakruojis district (55.978° N, 23.806° E), was sampled in October 2011 (8 individuals), and site 3, in the Žagarė Regional Park, Joniškis district (55.286° N, 23.207° E), was sampled in September 2014 (29 individuals). A total of 61 individuals were randomly selected for genetic analysis from all *A. oeconomus* captures.

As the captures of *A. oeconomus* in Lithuania were rather accidental, three sites with the highest trapping rates were selected for the study period to represent established populations. Habitats in the investigated sites were as follows: flooded meadows in Site 1, a natural mowed meadow in Site 2, and wet forests, forest wetlands, and shrubby meadows in Site 3 (Figure 1). As is shown by the maps, habitats in all three sites of small mammal trappings were fragmented and separated by natural (rivers, forests) and anthropogenic (roads, settlements, agricultural areas) barriers.

The trapped small mammals were kept in a refrigerator. At dissection, the hearts of the individuals were placed in vials and refrigerated in 70% ethanol until analysis.

The study was conducted in accordance with Lithuanian and European legislation on the protection of animals and approved by the Animal Welfare Committee of the Nature Research Centre, protocols No. GGT-7 and GGT-8. Further details are presented in the back matter.

### 2.2. DNA Isolation, PCR, and Sequencing 

Genomic DNA from *A*. *oeconomus* hearts was isolated using the universal salt extraction method [34] and diluted in 400 µL of nuclease-free water. The DNA concentration was determined using a NanoPhotometer^®^ P-300 spectrophotometer (Implen, Munich, Germany) and the samples were diluted to a final DNA concentration of 50 ng/µL. Partial fragments of the mtDNA *cytb* gene and the control region were used for the genetic characterization of *A*. *oeconomus* samples. The amplification of these two fragments was carried out by PCR using Micr-2L/Micr-2R and Pro+/MicrF primer pairs as described previously [6]. The quality of amplified fragments was evaluated using 1.5% agarose gel electrophoresis. To eliminate unincorporated nucleotides and primers, the PCR-obtained products were purified with the help of ExoI and FastAP enzymes (Thermo Fisher Scientific Baltics, Vilnius, Lithuania). The bidirectional sequencing was performed with the Big-Dye^®^ Terminator v3.1 Cycle Sequencing Kit (Thermo Fisher Scientific Baltics, Vilnius, Lithuania) and the 3500 Genetic Analyzer (Applied Biosystems, Foster City, CA, USA) according to the manufacturer’s recommendations. The resultant DNA sequences were manually edited to replace ambiguously placed nucleotides. The 697 bp *cytb* and 420 bp control region sequences of all sampled animals (61) were deposited in GenBank under accession numbers OR806886–OR806889 and OR806890–OR806894, respectively. All of the above sequences obtained in our study were used in each of the data analyses described in Section 2.3.

### 2.3. Phylogenetic Analyses

The phylogenetic analyses were carried out to identify which populations are closest to *A*. *oeconomus* collected in Lithuania. We also aimed to determine whether the partial *cytb* and control region sequences could be used to distinguish phylogenetic lineages of *A*. *oeconomus*. All sequences of analyzed genetic markers available in GenBank were used for phylogenetic investigations. 

For *cytb* analysis, 341 sequences were retrieved from GenBank (AB372193–AB372207 [35], AY219981–AY220045 [19], DQ452134–DQ452142 [14], FJ986325–FJ986326 [36], GU954319, GU987116 [37], KP190236–KP190237, KP326574 [38], KP684101–KP684121 [27], MF099520–MF099521, MF099544–MF099546, MF099577, MF099579–MF099581 [39], and AY305050–AY305263 [40]). Some of the sequences obtained from GenBank, namely AY219983, AY219986, AY219987, AY219989–90, AY219995, AY220007, AY220010, AY220014, AY220025, AY220027–9, AY220032, AY220036–7 [19], DQ452135, DQ452137, DQ452142 [14], KP684101–9, KP684111–4, KP684116, and KP684118 [27], were identified in 2–122 voles, and all remaining sequences were detected once. It should be noted that one of the sequences, AY220011, was previously obtained from *A. oeconomus* collected in the southern part of Lithuania (Žuvintas Strict Nature reserve) [19]. Most of the sequences studied originated from *A*. *oeconomus* captured in Finland, Sweeden, Norway, Poland, Canada, the USA, central Asia, and the Beringia of the Russian Federation. In addition, some of examined sequences were from Belarus, Hungary, Slovakia, the Netherlands, Mongolia, and China. The length of the *cytb* fragment being compared was 697 bp.

Overall, 267 control region sequences [11,40] were retrieved from GenBank (AY305050–AY305263, HM135795–HM135812, HM135907–HM135943) and compared with those determined in the present study. A major part of sequences was determined for *A. oeconomus* collected in Beringia, covering eastern Siberia and northwestern North America. Furthermore, some sequences were identified in individuals from Central Asia, Finland, Norway, the Tver region of the Russian Federation (not far from Moscow), and Austria. It should be noted that *A. oeconomus* from Poland were not characterized with the control region. Since the compared sequences were of different lengths, and started and ended at different nucleotide positions, the 378 bp overlapping control region fragments were used for data analysis. 

Apart from the current study, only in one study [40] were *cytb* and control region sequences of the same individuals determined (AY305050–AY305263). With the exception of three sequences (AY305161–3), all other sequences originated from *A*. *oeconomus* collected in Asia and North America. Therefore, phylogenetic analysis based on pooled *cytb* and control region data was not performed.

Haplotypes of *cytb* and the control region were ascertained with a help of FaBox v. 1.5 [41]. Multiple sequence alignments were generated using the ClustalW algorithm incorporated in the MEGA7.0.26 software [42]. The nucleotide substitution models with the best fit to the analyzed data were selected in MEGA7 on the basis on the calculated minimum values of the Bayesian Information Criterion. The initial phylogenetic analyses were performed using the neighbor joining (NJ) method [43] and the Tamura–Nei substitutions model [44] to establish the haplotypes that are most closely related to those identified in the present study. All sequences of examined mtDNA fragments available in GenBank were used for NJ phylogeny. Subsequently, the phylogeny of haplotypes selected by NJ analysis was reconstructed using a maximum likelihood (ML) method [45]. The bootstrap method with 10,000 replicates was used to evaluate the robustness of the suggested phylogeny. The NJ and ML phylogenetic tree were constructed with a help of MEGA7. The haplotype network was calculated using the median joining (MJ) method [46] implemented in NETWORK 10.2.0.0 software (https://www.fluxus-engineering.com/sharenet.htm, accessed on 2 October 2023).

### 2.4. Population Genetic Analysis

To assess genetic variability and the population structure of *A*. *oeconomus* sampled in the present study, inter-population genetic analyses were carried out. For this purpose, we also compared the genetic diversity and divergence of *A*. *oeconomus* from Lithuania with the most closely related populations of this species. 

The parameters of intraspecific genetic variability, i.e., the number of segregating sites (*S*), the number of haplotypes (*h*), the average number of nucleotide differences (*K*), the haplotype diversity (*Hd*), the nucleotide diversity (*π*), and the standard deviation (*SD*) for the last two indexes were assessed with a help of DnaSP v. 6 software [47].

Values of Tajima’s D neutrality test [48] were determined using DnaSP v. 6. Pairwise Φ_ST_ values indicating the level of genetic differentiation were evaluated using Arlequin v. 3.5.2.2 [49]. The statistical significance of Φ_ST_ values was tested by 10,000 permutations at the 95% confidence level. GenAlEx v. 6.502 [50] was employed to perform the principal coordinate analysis (PCoA) using Nei’s genetic distance [51].

The phylogenetic and population genetic data analyses described above were applied separately for both genetic markers: the *cytb* and the control region. Moreover, for the analysis of the intraspecific genetic variability, the neutrality statistics, and the PCoA calculations, pooled *cytb* and control region data were also used.

## 3. Results

### 3.1. The Origin of Lithuanian Alexandromys oeconomus 

The analysis of 61 partial 697-base-pair *cytb* sequences of *A. oeconomus* from Lithuania showed the existence of four haplotypes. However, based on the 420 bp control region comparison, five individual haplotypes were ascertained from the 61 sequences analyzed. The identified haplotypes differed by up to two single nucleotide polymorphisms (SNPs) within the control region and up to three SNPs within *cytb*. Five control region haplotypes defined in the present study were newly identified for *A. oeconomus*, and they differed by as many as three SNPs compared to other control region sequences detected in other countries for this vole species. 

**Figure 2:**
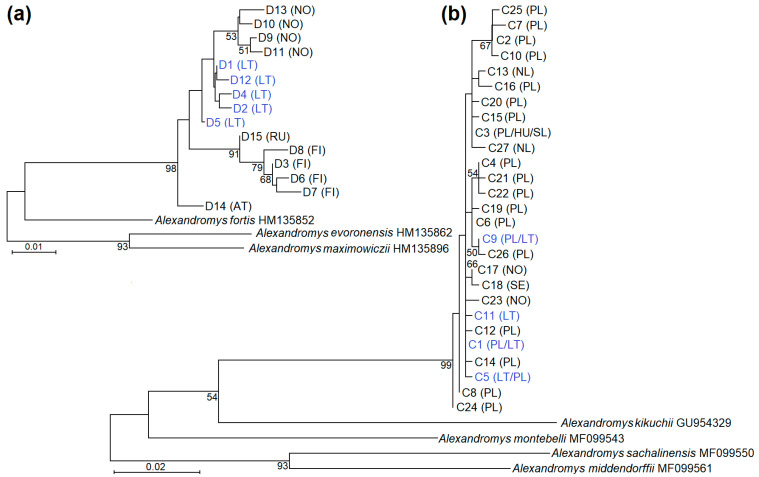
The ML trees of *A. oeconomus* based on control region (**a**) and *cytb* (**b**) sequences and rooted in other closely related *Alexandromys* species. Haplotypes detected in this work are shown in blue. Only haplotypes most closely related to those identified in Lithuania were compared. The selection of haplotypes for ML trees was based on the results of NJ analyses (Figure A1 and Figure A2). The HKY + G evolutionary nucleotide substitution model [42] was selected for both analyses (control region and *cytb*). The figures next to branches show bootstrap values higher than 50. The haplotypes identified in this study are indicated in blue. AT—Austria, FI—Finland, HU—Hungary, LT—Lithuania, NL—The Netherlands, NO—Norway, PL—Poland, RU—The Russian Federation, SE—Sweeden, SL—Slovakia.

**Figure A1:**
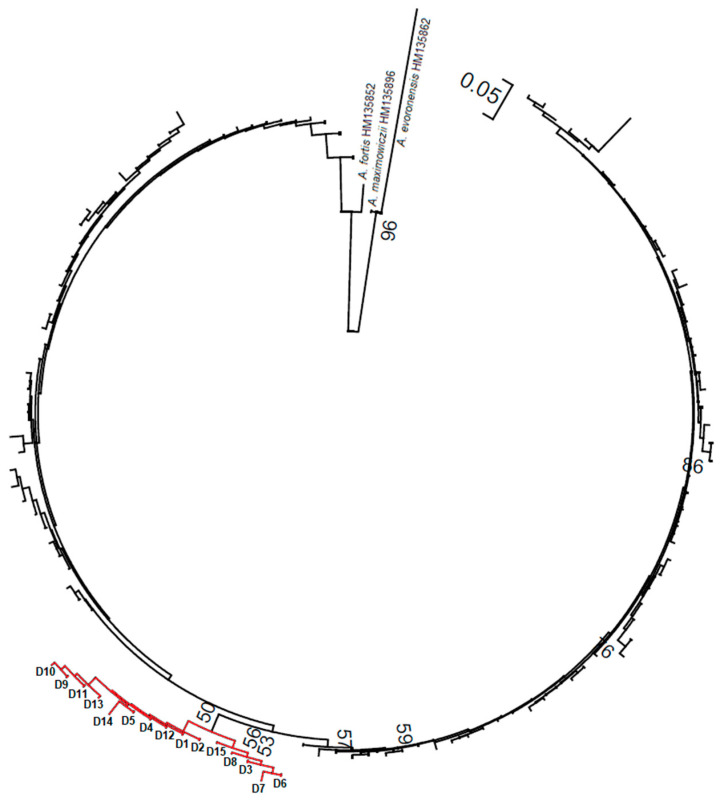
The circle phylogenetic tree of *A. oeconomus* based on control region sequences and constructed using NJ method. All sequences of the control region fragment analyzed from *A*. *oeconomus* available in GenBank were used. The figures next to branches show bootstrap values higher than 50. Fifteen haplotypes determined in Europe (D1–D15) formed separate cluster. Meanwhile, 254 sequences (AY305050–9, AY305061–160, AY305164-–0, AY305182–263, HM135803–12, HM135907–27, HM135931–43) attributed to 104 haplotypes were identified for voles collected in Asia and North America.

Overall, 119 haplotypes were defined for the studied control region sequences. In the preliminary NJ tree, all 15 control region haplotypes determined in Europe (Finland, Norway, Austria, the Tver region of the Russian Federation, and Lithuania from the current study) were placed into one cluster with a low (53) bootstrap support value (Figure A1). These haplotypes were named D1–D15. The GenBank accession numbers, country of origin, and frequencies of these haplotypes are listed in Table A1. Notably, the resolution power of the control region NJ tree was too low to discriminate between haplotypes identified in Central Asia and Beringia. The ML analysis showed that *A. oeconomus* haplotypes from Lithuania did not mix with haplotypes found in other countries (Figure 2a). The grouping of haplotypes from Finland and the Tver region of Russia was supported by a high and significant bootstrap value (91). In addition, four haplotypes detected in Norway were placed into one cluster with a low support value (53), while haplotype found in Austria formed a separate branch.

**Figure A2:**
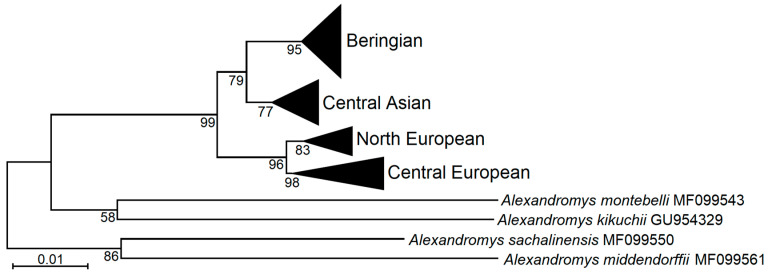
The NJ tree of *A. oeconomus* based on *cytb* sequences. All sequences of the *cytb* fragment analyzed from *A. oeconomus* available in GenBank were used. The figures below branches demonstrate bootstrap values higher than 50%. A total of 27 haplotypes (C1–C27), including C1, C5, C9, and C11, identified in the current study were placed into Central European phylogenetic group. Overall, 185 sequences (AB372197–207, AY220021–45, AY305050–160, AY305164–200, MF099546) that were assigned to 62 haplotypes established in *A. oeconomus* from North America and the Russia Far East were clustered in the Beringian lineage. Thirty-nine haplotypes (AB372193–6, AY220015–20, AY305201–63, FJ986326, KP190236–7, KP326574, MF099520–1, MF099544–5, MF099577, MF099579–81) defined in *A. oeconomus* from Russia, Mongolia, and China were combined in the Central Asian phylogenetic group. Finally, 26 haplotypes representing AY219981–AY220002, AY305161–3, DQ452134–42, and FJ986325 sequences originating from Belarus, Finland, Norway, Russia, and Sweden were assigned to the North European group.

The phylogenetic NJ analysis showed that 154 identified *cytb* haplotypes were grouped into four lineages: Beringian, Central Asian, North European, and Central European (Figure A2), as classified by C. Brunhoff et al. [19]. The classification of haplotypes into the four phylogenetic groups was supported by significant bootstrap values. Furthermore, the grouping of the European haplotypes of *A*. *oeconomus* was strongly supported (bootstrap value of 96). The four haplotypes identified in this study were placed into the Central Asian phylogenetic group together with 23 haplotypes identified in Poland, Hungary, Slovakia, the Netherlands, Norway, and Sweden. The following haplotypes were named C1–C27 (Table A1). Notably, all 21 haplotypes detected in Poland, all of those detected in Hungary, Slovakia, and the Netherlands, 2 out of 25 from Norway, and 1 of four from Sweeden were placed together with those from Lithuania. The other *cytb* sequences from Norway, Sweden, Finland, Belarus, and Russia were placed into the Northern European clade. In the current study, we identified C1, C5, C9, and C11 haplotypes. The first three haplotypes were also observed in Poland (Table A1), while the C11 haplotype was identical to that previously found in southern Lithuania [19]. Thus, all four *cytb* haplotypes defined in the present study were previously detected at other sites. Based on ML analysis, the clustering of C1–C27 haplotypes was not well defined, as clustering of only four clades was supported by 50–67 bootstrap values (Figure 2b). In summary, the studied animals captured in Lithuania were genetically closest to *A*. *oeconomus* collected in Poland. 

**Figure 3:**
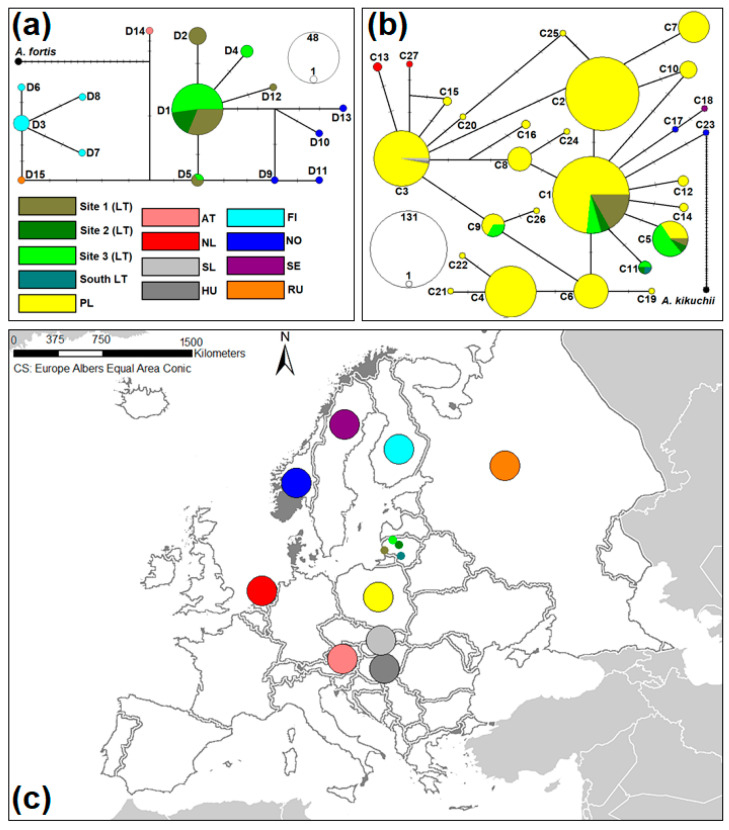
Haplotype network of *A. oeconomus* based on control region (**a**) and *cytb* (**b**) haplotypes. The area of the circle corresponds to the frequency of the haplotype. Dashes show mutational steps. The different colors represent countries in which the voles were caught (**c**). Sites 1–3 correspond to locations in Lithuania displayed in Figure 1. AT—Austria, FI—Finland, HU—Hungary, LT—Lithuania, NL—The Netherlands, NO—Norway, PL—Poland, RU—The Russian Federation, SE—Sweeden, SL—Slovakia.

Of the five control region haplotypes found in Lithuania, the most common D1 haplotype was found in 78.7% of the samples, present in all three sampling sites. Other haplotypes differed from D1 haplotype by only one mutational step (Figure 3a). Based on the *cytb* haplotype network, the two most common haplotypes in Lithuania, C1 and C5, were found in 88.5% of individuals. Both haplotypes were detected in all three sampling sites examined (Figure 3b); C1 and C5 were identified in 35 and 19 animals, respectively. However, the ratio of haplotypes clearly differed in terms of sampling sites. The C1 haplotype prevailed in the western part of Lithuania (Site 1), while C5 dominated in the northern part of the country (Site 3). The frequency of C11 was four, and this haplotype was observed only in Lithuania (Site 2, Site 3, and the southern part of Lithuania). Both C5 and C11 differed from the most common haplotype by a single mutational step, whereas C9 differed from C1 by two mutational steps. C9 was identified in four individuals trapped in Site 3, located in northern Lithuania. From the mutational viewpoint, intermediate haplotypes between C1 and C9, i.e., C3 and C6, were common in Poland. 

### 3.2. The Genetic Variability of A. oeconomus from Lithuania

Based on *cytb*, relatively low genetic variability (*K *= 0.80011, *Hd *= 0.589 ± 0.047, and *π* = 0.00115 ± 0.00017) was determined for Lithuanian samples of *A. oeconomus* in comparison to those established for animals collected in Poland (*K *= 2.80442, *Hd *= 0.833 ± 0.008, and *π* = 0.00402 ± 0.00009) (Table 1). Significantly higher values of genetic variability were estimated in animals sampled in the Northern European phylogenetic group (*K *= 5.68499, *Hd *= 0.958 ± 0.014, and *π* = 0.00816 ± 0.00071) than in animals collected in Lithuania and Poland. The low genetic variability of *A. oeconomus* from Lithuania, as compared to the Northern European samples, was also confirmed by the data of the control region. In the present study, *cytb* and the control region fragments analyzed showed similar nucleotide diversity, while greater haplotype diversity was found in the *cytb* gene. The Tajima’s D values obtained were insignificant for all samples, indicating the neutral evolution of the vole species examined.

Of the three Lithuanian samples of *A. oeconomus* studied, the highest intraspecific genetic variability in terms of *K*, *Hd*, and *π* was found at Site 3, when the data from both genetic loci were combined (Table 1). At the control region, no genetic variation was observed in Site 2, as all eight individuals had the most common haplotype D1 (Figure 3a). Very low genetic variability (*Hd *= 0.159 ± 0.094, *π *= 0.00023 ± 0.00014) was estimated in Site 1 within *cytb*.

### 3.3. The Inter-Population Genetic Comparison of A. oeconomus from Lithuania

**Figure 4:**
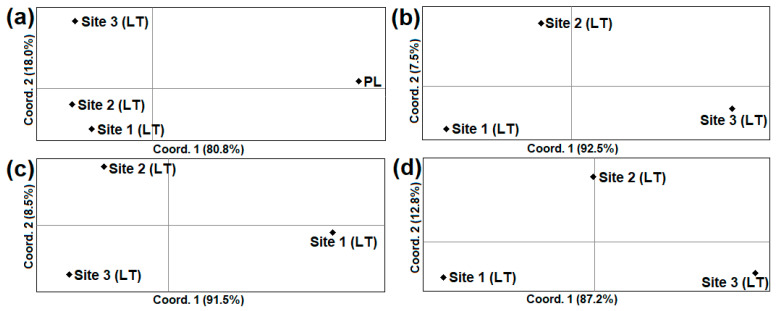
The principal coordinate analysis (PCoA) of *A. oeconomus* using Nei’s genetic distance of *cytb* (**a**,**b**), control region (**c**), and combined data of *cytb* + control region (**d**) sequences. LT—Lithuania, PL—Poland.

Lithuanian samples of *A. oeconomus* were genetically differentiated from samples collected in Poland (Φ_ST_ = 0.16660–0.20425; *p* < 0.05) and Northern Europe (Φ_ST_ ≥ 0.52409; *p* < 0.001) (Table 2). Average (Φ_ST_ = 0.12513, *p* < 0.001) and high (Φ_ST_ = 0.22077, *p* < 0.001) genetic differentiation was determined between Lithuanian Site 1 and Site 3 in the control region and *cytb*, respectively. The genetic divergence between the Lithuanian and Polish populations of *A. oeconomus* was also confirmed by PCoA analysis (Figure 4a). Comparing the Lithuanian samples, the largest genetic differences at both mtDNA loci were observed between Sites 1 and Site 3 (Figure 4b–d).

## 4. Discussion

### 4.1. Distribution of Alexandromys oeconomus

Regardless of whether Northern Europe was colonized by the Mediterranean populations [2] or from other refugia [9], the phylogeography of *A. oeconomus* in higher latitudes was strongly influenced by late Quaternary geological and climatic events [19]. Dispersal is thought to occur through hydrogenic habitats, so landscape origin may influence the genetic diversity of populations [52].

Why is *A. oeconomus* currently so widespread? The species primarily inhabits a variety of habitats, such as floodplain meadows and reedbeds [29,53], wet forests, and swamps [52]. However, the species is able to survive (or adapt) to anthropogenic habitats such as fruit orchards [54] and agricultural land [18,21], using the remaining areas of marshland as a refuge [55]. They reproduce intensively, producing several litters [53], and litter size increases from south to north across their range [33]. In Lithuania, the litter size and the number of litters per year of *A. oeconomus* is small and therefore similar to other populations in the southern part of its geographical range [33].

The herbivorous diet allows species to use a wide range of food resources [56], which contributes to survival in different habitats. *Alexandromys oeconomus*, especially males, are able to disperse over long distances, but survival is low [57].

### 4.2. Low Genetic Variability of the Lithuanian Alexandromys oeconomus Population

We found that the genetic diversity of *A*. *oeconomus* in Lithuania is quite low, with only five *cytb* haplotypes, all of which have previously been detected in Poland or southern Lithuania (Table A1). 

We have identified five new control region haplotypes. The non-detection of these haplotypes in the past is most likely due to the fact that *A. oeconomus* from Poland has not been tested using the D loop. In Lithuania, low haplotype diversity (in *cytb Hd* = 0.577 ± 0.047; in the control region *Hd* = 0.372 ± 0.076) and nucleotide diversity (in *cytb* π = 0.00112 ± 0.00017, in the control region π = 0.00107 ± 0.00024) was observed at both mtDNA loci (Table 1), compared to *A. oeconomus* from Poland and Northern Europe (*Hd *= 0.833 ± 0.008–0.958 ± 0.014; *π *= 0.00402 ± 0.00009–0.01552 ± 0.00252). The relatively low genetic variability of Lithuanian populations of *A. oeconomus* found in this study should be considered an exception in the European part of the species’ range. Notably, high genetic diversity in mtDNA has been found in *A. oeconomus* collected in different regions of Europe [19,22,27], including modern populations on the Norwegian islands, living in harsh climatic conditions [14]. High mtDNA genetic variability is also characteristic of *A. oeconomus* accessions from Central Asia, Northeast Asia, and Alaska [19,23,34].

Furthermore, microsatellite markers have revealed relatively high genetic variability in central and northern European samples of *A. oeconomus* [16,17,23]. For instance, an analysis of 20 microsatellite loci conducted over a brief period in the Polish Bialowieza population of *A. oeconomus* revealed a high expected heterozygosity, a crucial indicator of genetic diversity, ranging from 0.72 to 0.78 [23]. Additionally, microsatellite analyses demonstrated similarly elevated genetic diversity in populations from Austria, Finland, Germany, Hungary, the Netherlands, Norway, and Slovakia [16,17,18].

In Lithuania, the genetic variability among vole species, besides *A*. *oeconomus*, has also been assessed in *M. agrestis* [6]. Using the same PCR primer pairs as in this study, considerably higher genetic variability values were estimated in *M. agrestis* within *cytb* (*Hd *= 0.841 ± 0.038, *π *= 0.00694 ± 0.00039) and the control region (*Hd *= 0.890 ± 0.021, *π *= 0.01147 ± 0.00070). Therefore, the diminished genetic variability observed in *A. oeconomus* from Lithuania is likely associated with the ecological characteristics of the studied species. Populations of *A. oeconomus* have formed over the last 70 years [9,23], and the decline in genetic variability can be attributed to the founder effect. It should be highlighted that within the control region, only a single haplotype was observed in Site 2 (Table 2). However, very low genetic variability was estimated in Site 1 within *cytb* (*Hd *= 0.159 ± 0.094, *π *= 0.00023 ± 0.00014). The reduced genetic variability in Site 1 can be explained by genetic drift due to the huge fluctuations in population size in this sample. The local population of *A. oeconomus* in southwest Lithuania (Site 1) has suitable habitats, including the preferred habitat of reedbeds [58], but they experience regular spring floods [30]. These floods contribute to the habitat’s suitability for voles by altering microtopography, enhancing food quality through increased plant biomass, and providing cover through taller vegetation. A similar positive impact is observed in the tundra ecosystem due to fires [59].

### 4.3. The Isolation, Spread, and Genetic Diversity of Alexandromys oeconomus

The absence of *A*. *oeconomus* in most of Latvia and Estonia has been attributed to the species spreading through Lithuania [29]. Currently, there is no alternative explanation for the absence of this species in two of the Baltic States. In Lithuania, the species initiated its spread from the southwest, likely originating from northeastern Poland [25,29]. Over the past 70 years, the species has expanded to northeastern Lithuania and reached Latvia [15].

The phylogenetic and haplotype network results of this study (Figure 2 and Figure 3) confirm that Lithuanian *A. oeconomus* originates from northeastern Poland. Three *cytb* haplotypes (C1, C5, and C9) were shared by *A. oeconomus* sampled in Poland and Lithuania. These haplotypes were detected in northeastern Poland, see locations 1–14, 16, 18, 20, and 21 in Figure 1 from Janczewicz et al. [27]. In more detail, C1, the most commonly identified haplotype in Lithuania, was detected at 15 different sites in Poland. Meanwhile, C5, with a detection rate of 51.7% at Site 3, was also detected at three sites in Poland—marked as locations 2, 11, and 16 in Figure 1 from Janczewicz et al. [27].

The different proportions of haplotypes observed in the three Lithuanian sites studied resulted in significant genetic differentiation between Lithuanian sites 1 and 3 (Table 2). The genetic divergence of these two sites was also confirmed by PCoA analysis (Figure 4). Importantly, despite the short period of separation from the Polish population, genetic differentiation (Φ_ST_ = 0.16660–0.20425; *p *< 0.001) was observed between Lithuanian and Polish samples of *A*. *oeconomus*.

Being associated with wetlands and humid habitats [52,53,55], *A. oeconomus* is expected to lack the most favorable habitats within agricultural landscapes. The remaining fragments of preserved wetlands function as refugia [55], sustaining relatively small populations. Such small populations are susceptible to genetic drift [60], leading to a reduction in genetic variability over time. The isolation of these small populations further restricts gene flow, fostering the development of populations with distinctive genetic variations [61]. The situation is further complicated by the fragmentation of hydrogenic habitats [52].

Genetic isolation also operates at a local scale [15], accentuating the impacts of climate-change-related alterations to wetland ecosystems and the desiccation of wet grasslands. Lopucki et al. demonstrated that gene exchange between local populations of *A. oeconomus* is possible even when separated by several kilometers of unfavorable habitat, but anthropogenic barriers may exert a stronger isolating effect [10]. Population fragmentation is also prevalent in other countries, such as Hungary [18] and Austria [62]. In our study, the two northern sites are situated in an anthropogenized landscape characterized by intensive agriculture and fragmented wet habitats. The dispersal potential of *A. oeconomus* may not be sufficient to counteract the genetic effects of isolation [10,57].

Hence, we concur with Domínguez et al. [63] that short-distance dispersals extend a species’ range but often lead to a loss of genetic diversity. This likely occurred during the spread of *A. oeconomus* in Lithuania. While water barriers showed no significant impact on the species, anthropogenic barriers might have had genetic effects, as suggested by García et al. [64].

## 5. Conclusions

Using mtDNA *cytb* and control region sequences, we observed a relatively low genetic variability in *A. oeconomus* specimens sampled in Lithuania compared to other European populations. The analysis of sample animals from three distinct sites in the country revealed the existence of four *cytb* and five control region haplotypes. These findings validate a prior ecological hypothesis suggesting the migration of *A. oeconomus* to Lithuania from northeastern Poland. Additionally, the *A. oeconomus* samples obtained in Lithuania exhibited genetic differentiation from populations in Poland and Northern Europe.

## Figures and Tables

**Table 1:** The intraspecific genetic variability and neutrality test of *A. oeconomus*.

Sample	*n*	*S*	*h*	*K*	*Hd* ± SD	*π* ± SD	Tajima D
*cytb*							
Lithuania	62	4	4	0.80011	0.589 ± 0.047	0.00115 ± 0.00017	−0.13146
Lithuania, present study	61	4	4	0.78033	0.577 ± 0.047	0.00112 ± 0.00017	−0.18937
Lithuania, Site 1	24	1	2	0.15942	0.159 ± 0.094	0.00023 ± 0.00014	−0.68111
Lithuania, Site 2	8	2	3	0.67857	0.607 ± 0.164	0.00097 ± 0.00032	−0.44794
Lithuania, Site 3	29	4	4	1.14286	0.655 ± 0.065	0.00164 ± 0.00029	0.31626
Poland	448	19	21	2.80442	0.833 ± 0.008	0.00402 ± 0.00009	−0.03417
Central Europe ^1^	518	26	27	2.72261	0.842 ± 0.008	0.00391 ± 0.00008	−0.80183
Northern Europe ^2^	44	38	24	5.68499	0.958 ± 0.014	0.00816 ± 0.00071	−1.20614
Overall	562	60	51	4.64387	0.865 ± 0.007	0.00666 ± 0.00006	−1.32441
control region							
Lithuania	61	4	5	0.40328	0.372 ± 0.076	0.00107 ± 0.00024	−1.14912
Lithuania, Site 1	24	3	4	0.63406	0.562 ± 0.092	0.00168 ± 0.00035	−0.53008
Lithuania, Site 2	8	0	1	–	–	0	–
Lithuania, Site 3	29	2	3	0.26108	0.254 ± 0.100	0.00069 ± 0.00028	−1.00859
Northern Europe ^3^	14	19	10	5.86813	0.890 ± 0.081	0.01552 ± 0.00252	−0.07419
Overall	75	22	15	2.52252	0.582 ± 0.007	0.00667 ± 0.00126	−1.33757
*cytb* + control region							
Lithuania	61	8	8	1.18361	0.754 ± 0.039	0.00106 ± 0.00012	−0.80927
Lithuania, Site 1	24	4	5	0.79348	0.656 ± 0.083	0.00071 ± 0.00013	−0.70896
Lithuania, Site 2	8	2	3	0.67857	0.607 ± 0.164	0.00061 ± 0.00020	−0.44794
Lithuania, Site 3	29	6	6	1.40394	0.761 ± 0.054	0.00126 ± 0.00019	−0.23334

^1^ Central Europe corresponded to *A. oeconomus* samples presented in Figure 2b and Figure 3b; ^2^ Northern European phylogenetic clade was determined using NJ analysis and it represents 23 individuals from Norway, 13 from Finland, 5 from European Russia, 2 From Sweeden, and 1 from Belarus; ^3^ the sample represent 8 animals from Finland, 4 from Norway, 1 from Austria and 1 from Tver region of Russia.

**Table 2:** Genetic differentiation of *A. oeconomus* samples. Pairwise Φ_ST_ values obtained based on *cytb* and control region sequences are presented below and above the diagonal, respectively. Statistically significant values are in bold.

	Site 1 (LT)	Site 2 (LT)	Site 3 (LT)	Poland	Northern Europe
Site1 (LT)		0.05900	**0.12513** **	–	**0.63653** **
Site 2 (LT)	0.06606		–0.02346	–	**0.52409** **
Site 3 (LT)	**0.22077** **	0.02509		–	**0.68879** **
Poland	**0.18365** **	**0.16660** *	**0.20425** **		-
Northern Europe	**0.77818** **	**0.72406** **	**0.77798** **	**0.82620** **	

LT—Lithuania, * *p* < 0.05, ** *p *< 0.001.

## Data Availability

This is ongoing research; therefore, unpublished data are not available publicly. All other data are available in the cited publications. The obtained different haplotypes of *cytb* and the control region are available via the GenBank database under accession numbers OR806886–OR806894.

## Appendix A

**Table A1:** Haplotypes, and their frequency, origin, and GenBank accession numbers. Sequences obtained in the present study are in bold.

Haplotype	Country	GenBank Acc. No.	Frequency
Control region			
D1	Lithuania	**OR806890**	48
D2	Lithuania	**OR806891**	6
D3	Finland	AY305161–63, HM135801–02	5
D4	Lithuania	**OR806892**	3
D5	Lithuania	**OR806893**	3
D6	Finland	HM135799	1
D7	Finland	HM135798	1
D8	Finland	HM135800	1
D9	Norway	HM135796	1
D10	Norway	HM135797	1
D11	Norway	HM135795	1
D12	Lithuania	**OR806894**	1
D13	Norway	HM135929	1
D14	Austria	HM135930	1
D15	Russia	HM135928	1
*cytb*			
C1	Poland	AY220010, KP684103, KP684111, KP684120	96
C1	Lithuania	**OR806886**	35
C2	Poland	KP684109	122
C3	Poland	AY220012, KP684101	68
C3	Hungary	AY220014	1
C3	Slovakia	AY220014	1
C4	Poland	GU987116, KP684104	60
C5	Lithuania	**OR806887**	19
C5	Poland	KP684105	10
C6	Poland	KP684112	26
C7	Poland	KP684107	22
C8	Poland	AY220013, KP684113	13
C9	Poland	KP684102	8
C9	Lithuania	**OR806888**	4
C10	Poland	KP684116	7
C11	Lithuania	AY220011, **OR806889**	4
C12	Poland	KP684114, KP684117	3
C13	Netherlands	AY220007	2
C14	Poland	KP684106	2
C15	Poland	KP684118	2
C16	Poland	KP684108	2
C17	Norway	AY220005	1
C18	Sweden	AY220003	1
C19	Poland	AY220008	1
C20	Poland	KP684119	1
C21	Poland	GU954319	1
C22	Poland	KP684121	1
C23	Norway	AY220004	1
C24	Poland	KP684110	1
C25	Poland	KP684115	1
C26	Poland	AY220009	1
C27	Netherlands	AY220006	1
